# Extrachromosomal circular DNA (eccDNA): from carcinogenesis to drug resistance

**DOI:** 10.1007/s10238-024-01348-6

**Published:** 2024-04-25

**Authors:** Zhaoxing Li, Daohai Qian

**Affiliations:** Department of Hepatobiliary Surgery, Yijishan Hospital of Wannan Medical College, Wuhu, China

**Keywords:** eccDNA, Cancer, Biogenesis, Drug resistance

## Abstract

Extrachromosomal circular DNA (eccDNA) is a circular form of DNA that exists outside of the chromosome. Although it has only been a few decades since its discovery, in recent years, it has been found to have a close relationship with cancer, which has attracted widespread attention from researchers. Thus far, under the persistent research of researchers from all over the world, eccDNA has been found to play an important role in a variety of tumors, including breast cancer, lung cancer, ovarian cancer, etc. Herein, we review the sources of eccDNA, classifications, and the mechanisms responsible for their biogenesis. In addition, we introduce the relationship between eccDNA and various cancers and the role of eccDNA in the generation and evolution of cancer. Finally, we summarize the research significance and importance of eccDNA in cancer, and highlight new prospects for the application of eccDNA in the future detection and treatment of cancer.

## Introduction

Cancer is a major public health problem that is associated with a high mortality rate and a poor quality-of-life in some cancer patients, thus representing a serious threat to human health [1, 2]. The occurrence of disease is associated with specific causes and mechanisms, and the development of cancer is no exception. Chromatin and its related epigenetic mechanisms in cells of the body can maintain specific gene expression patterns and cellular homeostasis under normal physiological conditions, so as to adapt to changes in various development and survival conditions. However, due to some abnormal genetic, environmental or metabolic stimuli and other factors, there may cause changes in the epigenetic environment of cells and chromatin distortion, thus causing cancer and other diseases [3]. The occurrence and development of cancer is not caused by a single factor; on the contrary, it is caused by an interaction between complex and diverse molecular mechanisms. Abnormal gene expression is the hallmark of cancer [4, 5] and one of the key driving factors of this disease. This includes changes in gene sequence, such as point mutation, chromosomal translocation, deletion and insertion, and changes in the gene copy number, such as gene amplification and other gene activation mechanisms [6, 7]; these events can promote large-scale DNA recombination [8], thus leading to cancer.

Extrachromosomal circular DNA (eccDNA) refers to circular DNA that originates from chromosomes, but is likely to be independent of chromosomal DNA once produced. Human cells have 23 pairs of chromosomes, some of which can be amplified in the DNA outside the chromatin under certain environmental conditions. Therefore, there is a special type of circular DNA molecules that are independent of the chromosome genome in cells, which are collectively referred to as eccDNA. These are separated from normal chromosomes and genomes, and form a single or double stranded closed circular DNA structure [9, 10]. In 1964, Alix Bassel and Yasuo Hoota identified eccDNA in the nucleus of pig sperm for the first time by electron microscopy; researchers named it double minutes (DMs) [11]. Subsequent research revealed that the characteristics and functions of eccDNA are increasingly being exposed to public view, and it is now clear that a wide variety of eccDNA is widely present in eukaryotic cells, with sizes ranging from hundreds of base pairs (bp) to several million bases (Mb). Based on source and size, eccDNA can be divided into mitochondrial DNA (MtDNA), episomes, double minutes (DMs) (100 Kb–3 Mb), telomere rings (T-rings), small polydisperse circular DNA (spcDNA) (100 bp–10 kb), and microDNA (100–400 bp) [12]. With increased research activity, the functions of eccDNA are gradually being reported. In addition to functions such as aging [13–15] and heterogeneity [16], researchers have found that the length of eccDNA is long enough to feature its own replication starting point, as well as to encode amino acids and form specific proteins to function, which have been detected in tumor tissues. For example, in non-small cell lung cancer, the eccDNA of PLCG2 is upregulated in NSCLC cells, resulting in phosphatidase Cγ2, as a transmembrane signal transduction enzyme, is highly expressed in NSCLC tissues and cells, thereby promoting the progression of NSCLC [17]; the amplification of DHFR gene can also enhance tumor resistance to MTX by increasing the production of DHFR [18]. Therefore, in cancer therapy, eccDNA often features oncogenes or genes associated with drug resistance [19–22]. These characteristics enable eccDNA to play a positive role in the progression of cancer, effectively promoting cancer progression. In this review, we discuss the biogenesis of eccDNA, its relationship with tumors and its role in tumor development, and provide prospects for its future clinical applications.

## Biogenesis of eccDNA

In the decades following the discovery of eccDNA, it has been found that the formation of eccDNA in human cells typically involves damage to chromosomal DNA and incorrect behavior through different DNA repair pathways [23]. Studies have shown that heterochromatin in the body is considered the guardian of the genome, which can protect the integrity and stability of the genome [24]. Therefore, it is speculated that one of the reasons for DNA damage may be due to the decreased protective ability of noncoding DNA against the genome [25]. Some studies have also found that transcription can induce the formation of eccDNA during the aging process of yeast cells [15]. Another study has shown that deoxyribonuclease 1 like 3 (DNASE1L3) can digest eccDNA outside the cell, thereby reducing the accumulation of eccDNA [26]. Researchers have performed in-depth studies relating to the mechanisms responsible for eccDNA and found that eccDNA can be produced by many different ways and different mechanisms. Following decades of research, several eccDNA formation models have been proposed, including the break–fusion–bridge (BFB) cycle model, chromosome fragmentation, the episome model, and the translocation–excision–deletion–amplification model [27–30].

### The break–fusion–bridge (BFB) cycle model

The break–fusion–bridge (BFB) cycle model is a common mechanism for gene amplification [31]. The break–fusion–bridge (BFB) cycle begins with double strand breaks (DSBs) in DNA. During the process of cell mitosis, the existence of dissociation factor (DS) can cause the chromosome at its location to break; this causes the loss of telomeres and some genes. The two ends of the broken chromosomes can fuse with each other to form a chromosome with double centromeres. During the later stage of cell division, the two centromeres are separated from each other and move to two stages due to the action of the spindle filament, thus forming a “chromosome bridge.” A late break in the chromosome can produce a telomere-deficient new end on one end, repeats on the other end, and triggers another round of amplification in the BFB cycle. Because the location and size of the break differ, the eccDNA generated is also random [32, 33].

### Chromosome fragmentation

Chromosome fragmentation is different from other models that need to be carried out gradually; some studies have shown that chromosome fragmentation is a one-time event, thus resulting in rearrangement of the cancer genome [34]. During this process, one or several chromosomes in the cell are broken, forming tens to hundreds of sequence fragments. During the process of late chromosome repair, these fragments can be arranged, joined, and cycled into eccDNA elements in a random order [35]. Researchers have identified this particular phenomenon in a variety of tumors, including pancreatic cancer, neuroblastoma, prostate cancer, pediatric medulloblastoma, and small cell lung cancer [36–40]. In small cell lung cancer, eccDNA has been shown to be associated with chromosome division and lead to amplification of the MYC oncogene [34].

### Episome model

The episome model is one of the classical models of eccDNA. During the process of DNA synthesis, cyclic DNA is generated through a DNA slippage and R-ring, cut and joined, and can then be amplified by the integration of other DNA components (such as promoters and enhancers) [41].

### Translocation–excision–deletion–amplification model

The translocation–excision–deletion–amplification model triggers the formation of translocation through the generation of multiple DSBs. Then, the fragments close to the translocation break point can be amplified, excised, and then cycled to produce eccDNA [42].

This model suggests that eccDNA is formed through multiple pathways and can be produced through one or several pathways, thus playing a key role at the cellular or whole-body level. Although the specific steps of eccDNA production have yet to be fully investigated and further studies are urgently needed, we have come to the conclusion that eccDNA is formed by the DNA fragments generated after chromosome breakage through connection, circulation, and other ways, and play an important role in the development of cancer.

## eccDNA and cancer

**Fig. 1:**
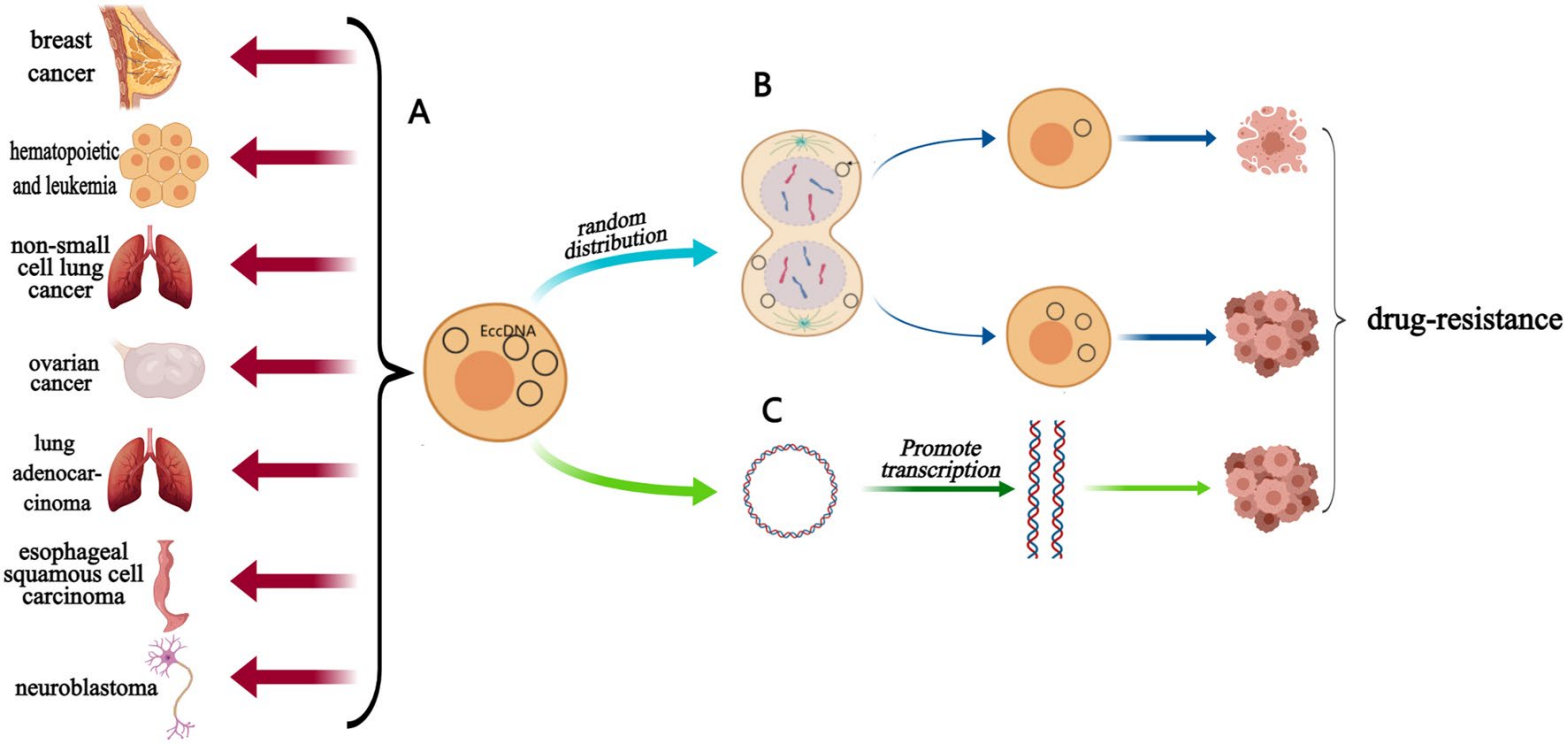
**A** eccDNA and known associated cancers. **B** eccDNA carries carcinogenic genes. **C** eccDNA increases the copy number of oncogenes

EccDNA is closely related to the occurrence and development of various cancers and affects the progression of cancer through various ways and functions (Table 1) [43]. These findings indicate that eccDNA may represent a new biological target and play an important role in the process of tumor detection and treatment (Fig. 1A).

**Table 1:** EccDNA and cancers

Types of cancers	EccDNA
Breast cancer	*ITGB7*
Acute myeloid leukemia (AML)	*NRAS, MCL1, EVI1, GATA2, WT1, PAK1, GLYATL1*
Non-small cell lung cancer (NSCLC)	PLCG2
High-grade serous ovarian cancer (HGSOC)	*DNMT1circle10302690-10302961*
Lung adenocarcinoma (LUAD)	*DOCK1, PPIC, TBC1D16, RP11-370A5.1*
Esophageal squamous cell carcinoma (ESCC)	The deletion or amplification of eccDNA
Neuroblastoma	MYCN gene

### eccDNA and breast cancer

Breast cancer (BC) is considered to be a systemic disease with inter-tumor and intra-tumor heterogeneity and a poor prognosis [44]. To date, BC is still one of the most common malignant tumors in the world and the leading cause of death in women [45, 46]. An earlier study [47] predicted that there would be 2.26 million new cases of breast cancer worldwide in 2021, thus accounting for 11.7% of the total number of new cancer cases worldwide. Therefore, research targeted towards breast cancer is a very important task for medical staff and scientific researchers. During this process, some researchers have found that eccDNA plays a certain role in the progression of breast cancer. One study [48] detected eccDNA in a specimen of BC and identified a total of 200 eccDNA genes that were mainly related to the PI3K-Akt signaling pathway and focal adhesion. With regard to biological processes (BPs), eccDNA was found to be mainly concentrated in actin, cytoskeletal recombination and synaptic guidance. In terms of molecular functions (MFs), eccDNA was found to be mainly enriched in sodium ion transmembrane transporter activity and metal ion transmembrane transporter activity. For cellular components (CCs), eccDNA was found to be mainly concentrated in transcriptional regulatory complex and focal adhesion. Among these 200 genes, the gene that encodes *ITGB7* is significantly enriched in cell–matrix adhesion and intramembrane localization in BPs, integrin binding in MFs, and cell-substrate binding and focal adhesion in CCs. Comparisons with normal samples showed that *ITGB7* was significantly upregulated in BC patients. This indicated that ITGB7 was closely related to the cause of BC. Research demonstrated that the expression of *ITGB7* in BC samples was significantly different when compared between stages 1, 2, and 3. Furthermore, analysis indicated that *ITGB7* may exert impact on the prognosis of BC patients in different menstrual states. Therefore, The eccDNA encoding *ITGB7* may be used as a prognostic indicator for BC patients, and is also of great significance for the individualized clinical treatment of BC patients.

### eccDNA and hematopoietic and leukemia

Previous research showed that eccDNA amplification does not occur in blood or normal tissues [49]. And some other studies have shown that double minutes (Dms) in acute myeloid leukemia (AML) and myelodysplastic syndrome (MDS) are associated with micronuclei, MYC or MLL amplification, complex karyotypes, monomeric karyotypes, TP53 deletions, and TP53 mutations [50, 51]. Researchers are also making great efforts to explore this area. One previous study [52] revealed the layout of eccDNA in normal hematopoietic and AML and found that the appearance of eccDNA was accompanied by the hematopoietic process of all cell types; the number of eccDNA gradually increased with the process of differentiation from original cells to terminal cells. In particular, the amount of microDNA ias proportional to the degree of cellular differentiation. This study also found that eccDNA also existed in AML, and that these eccDNAs may play an important role in the evolution of AML and normal hematopoietic cells. Compared with normal hematopoietic cells, AML-related genes have been shown to expressed at higher levels in eccDNA during the development of AML, including *NRAS, MCL1, EVI1, GATA2, WT1, PAK1*, and *GLYATL1*. Therefore, during the development of AML, the accumulation of eccDNA and oncogenes (*NRAS, MCL1, EVI1, GATA2, WT1, PAK1*, and *GLYATL1*) in eccDNA may contribute to the progression of AML.

### eccDNA and non-small cell lung cancer

Lung cancer is an extremely common cancer and a major cause of cancer-related death; recent reports indicated that approximately 350 people die from lung cancer every day worldwide [53]. In all classifications of lung cancer, non-small cell lung cancer (NSCLC) accounts for 80% of all lung cancer cases and has become the main pathological type of lung cancer [54]. Because of its low 5-year survival rate, the study of NSCLC is also extremely important. Previous studies [17] have found that eccDNA plays a certain role in promoting the progression of NSCLC. This study found that phosphatidase Cγ2 (PLCG2), as a transmembrane signal transduction enzyme, is expressed at high levels in NSCLC tissues and cells; furthermore, the eccDNA of PLCG2 is up-regulated in NSCLC cells. Therefore, this study suggested that PLCG2 can exist in the form of eccDNA in lung cancer; furthermore, PLCG2, as an oncogene, can promote NSCLC cell metastasis by enhancing mitochondrial function.

### eccDNA and high-grade serous ovarian cancer

High-grade serous ovarian cancer (HGSOC) is the most common and lethal subtype of epithelial ovarian cancer, accounting for 70% of all deaths from ovarian cancer [55, 56]. This is the most aggressive subtype of ovarian cancer, often accompanied by metastasis and a poor prognosis. Therefore, medical personnel and scientific researchers pay significant attention to this disease. Few studies have investigated the relationship between eccDNA and HGSOC, but a recent publication did propose a role for eccDNA in HGSOC. Researchers described the eccDNA expression profile of primary and metastatic tissues of HGSOC and identified a new eccDNA that was named *DNMT1circle10302690-10302961*. Compared with the primary tumors of HGSOC, the expression of this new eccDNA in metastatic tumors was significantly down-regulated. The findings of this study linked the reduction of eccDNA with a poor prognosis in patients with HGSOC and may be a reliable clinical therapeutic target for the HGSOC metastasis in the future [57].

### eccDNA and lung adenocarcinoma

In addition to the eccDNA that we mentioned in the previous article can promote the metastasis of NSCLC [17], some studies have also found that eccDNA is also closely related to lung adenocarcinoma (LUAD). One study [58] performed NGS analysis of eccDNA in the plasma of six patients with lung adenocarcinoma and compared the size distribution, chromosome origin, formation, and expression patterns of eccDNA in these six LUAD patients with six healthy people and four healthy pregnant women. Analysis showed that the frequency of the nine types of eccDNA in lung adenocarcinoma patients was higher than that in healthy controls, with four types of eccDNA (*DOCK1*, *PPIC*, *TBC1D16*, and *RP11-370A5.1*) were only expressed in patients with lung adenocarcinoma, thus indicating that this specific eccDNA exists in the plasma of patients with lung adenocarcinoma. This discovery makes it possible for these eccDNAs to be developed into a highly advanced biomarker in LUAD detection.

### eccDNA and esophageal squamous cell carcinoma

Of the many types of cancer, esophageal cancer ranks seventh in terms of global cancer incidence and sixth in terms of cancer-related mortality [59]. In the classification of esophageal cancer, esophageal squamous cell carcinoma (ESCC) accounts for 90% of all esophageal malignancies in the world, and as the main pathological type of esophageal cancer, is distributed in high-risk areas in China [60]. The molecular mechanism for the high incidence rate and poor prognosis of esophageal squamous cell carcinoma has not yet been fully clarified. But domestic and foreign researchers have never given up, and more and more research results have shown that there is an undeniable relationship between eccDNA and cancer. A previous study [61] also found evidence of a certain relationship between eccDNA and esophageal squamous cell carcinoma. Researchers have reported the expression changes of eccDNA in matched ESCC samples. The deletion or amplification of eccDNA in normal esophageal epithelium may play a role in the initiation of ESCC by promoting the expansion of oncogenes or the production of functional small regulatory RNAs, thus suggesting that functional eccDNA can also be considered as a new effective target for tumor therapy.

### eccDNA and neuroblastoma

Neuroblastoma is the most common solid extracranial tumor in children and can show significant clinical heterogeneity. Previous studies have shown that amplification of the MYCN oncogene is detected in 20–30% of all detected cases of neuroblastoma [62, 63]. Valent et al. [64] found that the MYCN gene could be amplified by eccDNA, which was especially evident in patients with chemotherapy-resistant advanced neuroblastoma. Therefore, the MYCN gene can be amplified with the help of eccDNA, thereby increasing the expression of this gene in neuroblastoma cells, thereby promoting the tumor progression.

## eccDNA and drug resistance

Cancer is one of the main causes of death in humans. For cancer, in addition to surgical treatment, chemotherapy is also one of the main treatment methods for cancer patients [65, 66]. However, cancer patients often experience drug resistance during chemotherapy. It is because cancer cells develop tolerance and resistance to chemotherapy drugs, leading to a decrease in the inhibitory effect of drugs on cancer cells during the treatment process [67]. Thus, drug resistance is one of the main reasons for the failure of clinical chemotherapy. Drug resistance in cancer can be divided into endogenous drug resistance and acquired drug resistance [68]. Endogenous drug resistance occurs prior to chemotherapy and refers to the ability of cancer cells without chemotherapy to survive the initial treatment due to preexisting genetic changes or cell states, while acquired drug resistance develops through the acquisition of new mutations [69]. Most cancer cell resistance is acquired, that is, it develops through new mutations during chemotherapy. Therefore, there are many cancer patients who, even with corresponding chemotherapy drugs available for treatment, have poor chemotherapy effects, and even patients in advanced stages of cancer do not have available chemotherapy drugs. Therefore, the in-depth study of tumor resistance to chemotherapy drugs has become an urgent problem to be solved. Over recent years, an increasing number of studies have shown that eccDNA is involved in the process of drug resistance in cancer treatment and may also promote the generation of resistance to tumor drugs by carrying oncogenes and increasing oncogene copy number [27, 70].

### eccDNA carries carcinogenic genes

Cancer is not a single gene mutation of a cell; rather, it is a dynamic process, starting from a cell with normal genes, which then undergoes proliferation and differentiation; this is accompanied by a large number of gene mutations and accumulation, such that the normal cells become cancer cells with significant proliferation. Most of these mutations occur during mitotic chromosome replication [71]. The accumulation of mutations during this process promotes tumor heterogeneity, which is also a key characteristic of tumor cells [72]. Since the inheritance of eccDNA is different from that of chromosomes, it does not follow the law of Mendelian inheritance. During the process of cell mitosis, eccDNA can be randomly distributed into daughter cells, such that the number of eccDNAs in daughter cells is not equal [45]; furthermore, some eccDNAs will carry multiple carcinogenic genes. Therefore, daughter cells with eccDNA gain an advantage in terms of proliferation. This pathway can increase the diversity of the genome, thus promoting the heterogeneity of the tumor, making it easier for tumor cells to adapt to different environments; this is conducive to development of tumors and their resistance to chemotherapy drugs. (Fig. 1B).

### eccDNA increases the copy number of oncogenes

EccDNA can play a role in oncogene expression by increasing gene copy number, thus making eccDNA one of the important forms of oncogene amplification [70]. Research has shown that eccDNA has the same complete domain as chromatin, although it does not have a high order compression state [20]. As a result, genes on eccDNA are transcribed more easily; furthermore, enhancers carried by eccDNA can also promote transcription [73]. Due to these characteristics of eccDNA, there is a risk of the overexpression of oncogenes. Moreover, eccDNA can carry a variety of different genes, including the carcinogenic genes and drug resistance genes of cancer cells. Under the action of these multiple mechanisms, eccDNA may also induce cancer cells to develop drug resistance through gene expansion (Fig. 1C).

## Research techniques and experimental methods of eccDNA

In the process of studying eccDNA, researchers have been constantly trying various experimental methods in order to explore the true face of eccDNA. In the past few decades of studying eccDNA, researchers have used research methods such as electron microscopy [22], Karyotype [20], 2D gel electrophoresis [74, 75], Southern blotting [74], Inverse PCR [76, 77], fluorescence in site hybridization (FISH) [20–22], density gradient centralization [78], etc., to explore the secrets of eccDNA. These technologies can locate, identify, and quantify eccDNA, but there are also limitations. Some shortcomings, such as the inability of electron microscopy, Karyotype, 2D gel electrophoresis and other technologies to provide sequence information of eccDNA; Technologies such as Southern blotting, reverse PCR, and fluorescence in situ hybridization (FISH) can only provide a small amount of sequence information; however, density gradient centralization is very time-consuming and labor-intensive. A high-throughput sequencing technology [79] has recently been developed, which has extremely high resolution and sensitivity, but the high cost makes this technology unable to be considered as the primary method by researchers. Therefore, in the subsequent research process, finding a new experimental technique that can effectively avoid these shortcomings is a challenge that researchers have to face.

## Challenges and opportunities

EccDNA has only been discovered for decades, but its existence and importance to the organism are undisputed. This discovery has provided a new direction for cancer research; that is, to study the specific relationship between eccDNA and cancer. In this article, we review existing research relating to eccDNA and its relationship with cancer, propose some challenges and future opportunities, and provide some ideas for future research on eccDNA. For example, although scientists have proposed four models for the biogenesis of eccDNA (the break–fusion–bridge (BFB) cycle model, chromosome fragmentation, the episome model, and the translocation–excision–deletion–amplification model), its biogenesis and cycle mechanisms have yet to be fully elucidated. There are also significant differences between eccDNA and chromosome DNA in key aspects such as location, topology, and biology; therefore, researchers urgently need to develop new research tools and experimental methods with which to explore the specific role of eccDNA in the pathogenesis of cancer [80]. Furthermore, due to differences in the production and expression of eccDNA in cancer cells, eccDNA can be investigated as a potential biomarker for detecting cancer in the future. Furthermore, eccDNA has been shown to be related to drug resistance in some diseases. Therefore, there is an urgent need to improve our understanding of eccDNA by performing new research studies. Providing further evidence to support the potential use of eccDNA as a new biological target to inhibit resistance to tumor drugs is a crucial and significant research focus. Similarly, it is important that we combine therapy with traditional chemoradiotherapy or classical anticancer drugs to improve the efficacy and synergy of tumor therapy; this is an important direction of future research for eccDNA.

## Conclusion

Since researchers first discovered the existence of eccDNA in 1964, with the joint efforts of researchers around the world, the mysterious veil of eccDNA has been gradually revealed, allowing people to gradually see its true face. In these 60 years, the mechanisms responsible for the biogenesis of eccDNA has been studied, and four models have been proposed, namely the break–fusion–bridge (BFB) cycle model, chromosome fragmentation, the episome model, and the translocation-excision-deletion-amplification model. Researchers have also reported abnormal oncogene amplification in eccDNA; this is prevalent in various human cancers and can lead to a high copy number and expression levels of oncogenes, thus promoting tumorigenesis and adaptive evolution. Furthermore, eccDNA is also an important driving force for resistance to cancer drugs and has a close relationship with the poor prognosis of cancer and the development of genetic heterogeneity. Therefore, a deep understanding of the structure, mechanism of action, and function of eccDNA can contribute to our research on cancer progression, providing new detection targets and treatment plans for cancer detection and treatment, which is of great significance for the treatment and prognosis of cancer patients. In future research, more in-depth exploration of eccDNA is needed to reveal the regularity and characteristics of eccDNA, in order to be applied for detecting and treating cancer.

## Data Availability

Not applicable.
